# Case of Intussusceptio Successfully Treated by Injections

**Published:** 1853-09

**Authors:** E. M. Light

**Affiliations:** Oregon


ART. III.—*Case of Intussusceptio successfully treated by Injections.*—By E. M. Light, M. D.

January 1st, 1 o'clock, P.M., visited Mr.———, aged about thirty, of bilious-sanguine temperament; who was convalescing from a mild attack of typhoid fever. I found him with pulse seventy-four, tongue moist and cleaning. Patient said he felt quite comfortable, with the exception of an indescribable sensation, not amounting to pain, in the middle hypogastric region. Ordered laxative of seidlitz powders. At nine in the evening was summoned to patient: found his pulse as before, skin moist, occasional vomiting, frequent desire to go to stool, and a steady increase of pain in hypogastrium. Ordered morphia in ¼gr. doses every thirty minutes. Eleven o'clock, P. M., no abatement of pain, pulse as before, but irregular, stomach very irritable, vomiting about every half-hour, with frequent inclination to go to stool. Pain more severe. Left Sulph. morphia in gr. doses to be repeated every thirty minutes, until ease was felt. Mustard sinapisms to stomach and abdomen, pediluvium, &c.

The morphia was continued until four grains were taken, and given after that in $\frac{1}{4}$gr. doses, every hour, until complete ease was obtained. At eight, A. M., Jan. 2, visited patient: found his pulse 100, tongue dry and furring, occasional emesis, wide awake, perfectly conscious, and quite easy so long as complete rest was had, but upon stirring even an extremity some cutting pain was felt as before. Ordered a syrup of rhubarb and Sulph. potassii, with aromatics, to be given every half hour as the stomach would bear.

Considering the case as one of intussusceptio, and having read in a previous number of your Journal of the treatment of such cases with enemas, I immediately proceeded to their exhibition, and filled his bowels well with warm water and sweet oil. The injections gave pain, emesis and desire to stool. During an attempt at vomiting the bowels gave passage to wind, accompanied by dark liquid evacuations, very fœtid, and from this time emesis ceased, and perfect ease was had.

Bowels continued to pass fœtid and sanious evacuations, with decided dysenteric symptoms which, however, yielded readily to injections of laudanum and cold water, with the rhubarb, as before. From this time patient convalesced rapidly and has remained well to this time.

Oregon, July 6th, 1853.

